# Origin of anomalous giant dielectric performance in novel perovskite: Bi_0.5−*x*_La_*x*_Na_0.5−*x*_Li_*x*_Ti_1−*y*_*M*_*y*_O_3_ (*M* = Mg^2+^, Ga^3+^)

**DOI:** 10.1038/srep12699

**Published:** 2015-08-04

**Authors:** Xiao Liu, Huiqing Fan, Jing Shi, Qiang Li

**Affiliations:** 1 State Key Laboratory of Solidification Processing, School of Materials Science and Engineering, Northwestern Polytechnical University, Xi’an 710072, China

## Abstract

Dielectric properties and dielectric relaxation behaviors of A/B sites co-substituted Bi_0.5_Na_0.5_TiO_3_ perovskite-type ferroelectrics are reported. The Bi_0.5−*x*_La_*x*_Na_0.5−*x*_Li_*x*_Ti_1−*y*_*M*_*y*_O_3_ (*M* = Mg^2+^, Ga^3+^) exhibits anomalous giant dielectric permittivity (*ε*’) of ~10^5^ under a heterogeneous constitution with easily discernible grain and grain boundary conductivity. The lone pairs substitution theory as well as extrinsic disorders are used to clarify the significant structural evolution and the origin of the dielectric performance. A bigger free volume promotes the anomalous relaxation between oxygen sites, and the polarization direction on the nanoscale deviates from the average polarization direction at its ferroelectric state. Furthermore, no obvious phase transition indicates the considerable static substitutional disorder at the Bi/Na sites, which facilitates delocalized conduction of oxygen ions in the intermediate temperature range.

Solid oxide materials that exhibit ionic conductivity have attracted considerable attention in electrochemical applications, such as fuel cells, chemical sensors and catalysts^1^,^2^,^3^. Besides, they also play a predictive role in the design and improvement of dielectric properties of materials. Polarization occurs by the charge rearrangement when materials are applied with an external electric field. Oxide materials with high permittivity are always ascribed to the defect dipoles and space charge polarizations, where dipoles in the material reorient and charges accumulate at interfaces. Lanthanum doped BaTiO_3_ perovskites display colossal dielectric permittivity achieved by the activation of a great number of carriers and their trapping at the interfaces^4^. The cubic perovskite materials CaCu_3_Ti_4_O_12_ (CCTO) are found to have high permittivity, which can be attributed to an internal barrier layer capacitor mechanism (IBLC) with conductive grains and insulating grain boundaries in ceramics, while it is based on a non-ohmic electrode contact effect in single crystal^5^,^6^. The intrinsic contribution in CCTO originates from the dipolar effect related to disordered Ca/Cu sites^7^. Materials with high *ε*’ range from perovskite ferroelectrics, relaxors, binary oxides, and ionic conductors, in which the general characteristics are their high conductive grains. Both localized and delocalized conduction are bulk processes and give rise to the impressive geometric capacitance^4^,^5^,^8^,^9^,^10^,^11^,^12^,^13^.

Bi_0.5_Na_0.5_TiO_3_ (BNT) based ferroelectrics have seen a revival of research interest in recent years as an alternative to the toxic lead-containing piezoceramics^14^,^15^,^16^. BNT is perovskite-type ferroelectric with an A-site disorder structure. The 6*s*^2^ lone-pair electronic configuration of Bi^3+^ acts the same role as that of Pb^2+^ in PbZr_1−*x*_Ti_*x*_O_3_ (PZT). Normally, two major instabilities exhibit in BNT, rotations of the anion octahedra (tilting) and displacements of the cations (shifting), which are separately observed in other perovskite systems^17^,^18^. Additionally, BNT experiences a complicated phase transition sequence. Generally, it undergoes two structural phase transitions. A cubic phase (*Pm*3*m*) transforms into a tetragonal phase (*P*4*bm*) at *T*_c-t_ ≈ 540 ^o^C, where the transition corresponds to antiparallel cation displacements along [001] and *a*^0^*a*^0^*c*^+^ (Glazer notation) tilting of the oxygen octahedrons^19^,^20^. The tetragonal (*P*4*bm*) to either a rhombohedral (*R*3*c*) or a monoclinic (*Cc*) phase transition occurs at *T*_t-r_ ≈ 300 ^o^C, corresponding to *a*^−^*a*^−^*a*^−^ or *a*^−^*a*^−^*c*^−^ octahedral tilts and parallel cation [111] displacements. This transition is not well defined due to its diffuse nature and the crystal structure of BNT at room temperature is not yet settled. Various structural models have been proposed, such as the *Cc* phase, mixed *R*3*c* + *Cc* phase, *R*3*c* matrix with localized in-phase (+) tilted octahedral regions, etc.^21^,^22^,^23^,^24^,^25^ Interestingly, the application of a strong electric field can produce an irreversible transformation from *Cc* or mixed *R*3*c* + *Cc* to single *R*3*c* phase. When measured while drifting the temperature, the dielectric permittivity of poled BNT shows three dielectric anomalies. They are the shoulder with a strongly frequency dependent anomaly at ~200 ^o^C, the peak with a broad dielectric maximum at ~325 ^o^C, and the depolarization temperature at lower temperature ~190 ^o^C, above which the induced ferroelectric domains are dissociated into polarized nanoregions and double hysteresis loops are observed. BNT and its derivatives exhibit a maximum *ε*’ < 5000 at *T*_max_ to the best of our knowledge.

Recently, BNT with conductive grain have been reported to indicate the potential application as oxide ionic conductors in intermediate temperature SOFCs^26^. Thus, it is expected to exhibit potential high *ε*’ in this material as suggested above. The present paper first demonstrates and discusses the abnormally high *ε*’ of ~10^5^ and diffuse anomalies in BNT by modifying both A and B sites of ABO_3_ structure. Significant structural evolution with composition and temperature are studied, which is of great helpfulness to understand this BNT system since no literature is available for its special structure. The new BNT system is designed by introducing the lone pairs substitution conception as well as extrinsic disorders. As probed in anionic conductor Bi_2_O_3_, Bi^3+^ can adopt highly asymmetric anion surrounding with a stereo-chemically active lone pair directing toward oxygen vacancies^27^. Therefore, La^3+^ and Li^+^ atoms are employed as the obstructer from the oxygen sites depletion by lone-pair electron. Meanwhile, the low valence of Ga^3+^ or Mg^2+^ entering the Ti^4+^ site is to induce oxygen vacancies as the acceptor. We reveal considerable substitutional disorder at the Bi/Na sites that facilitates delocalized conduction of oxygen ions dominated by the soft covalent bonds. The frequency-dependent dielectric, impedance and electric modulus spectroscopies provide an evaluation of the steady state transport, and reflect the transient dielectric response resulting from localized displacements of the ions at high frequencies. Their structures are studied by using Raman spectra and XRD in a broad range of temperature from room temperature (RT) to 450 °C.

## Results and Discussion

**Figure 1:**
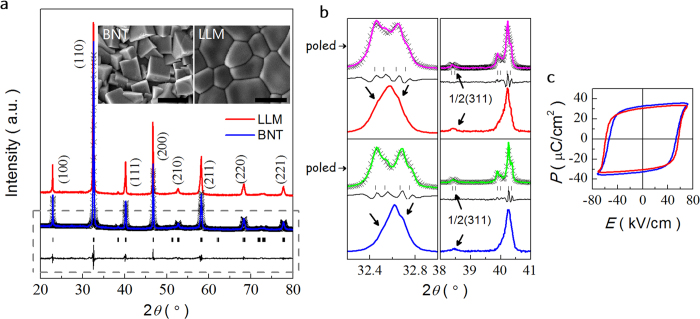
(**a**) Powder X-ray diffraction patterns of the sintered BNT and LLM with the Rietveld refinement using a *Cc* space group. The inset shows the SEM images of the BNT and LLM samples. The scale bar is 10 μm. (**b**) XRD details of the unpoled and poled BNT and LLM samples. XRD patterns of poled samples are refined using the space group *R*3*c*. (**c**) Polarization hysteresis of the BNT and LLM at electric field of 70 kV/cm at 1 Hz. The labeled *hkl* index are relative to the pseudocubic perovskite unit cell.

XRD patterns of the powder BNT and Bi_0.49_La_0.01_Na_0.49_Li_0.01_Ti_0.99_Mg_0.01_O_3_ (LLM) at RT are shown in Fig. 1a. It reveals a single perovskite structure, indicating that homogeneous solid solutions are formed. This is confirmed by the uniform and compact microstructure from the SEM micrographs of BNT and LLM. Fig. 1b illustrates the detailed XRD profiles of the pseudocubic peaks, {110}_*c*_, {111}_*c*_, and {200}_*c*_ of unpoled-crushed and poled-crushed specimens. Both the unpoled BNT and LLM patterns exhibit a monoclinic + rhombohedral phase (*Cc* + *R*3*c*) by the features of the three peaks in {110}_*c*_ and the peak splittings of {111}_*c*_. Here, the approximate *Cc* space group is used for the refinement rather than a *Cc* + *R*3*c* model considering the inaccuracy of the latter and the complexity of the phases for the unpoled BNT and LLM at RT^21^,^22^,^23^,^24^,^25^. Reasonably better quality is obtained by using the single *Cc* space group with *R*_p_ = 4.71%, 5.01% as compared with the *R*3*c* model. After poling, the strongest pseudocubic reflection {110}_*c*_ splits into a well-defined doublet with nearly equal intensities. The full pattern refinement is provided in Supplementary Fig. S1. A comparison of the profile shapes reveals the occurrence of field-induced phase transformation from *Cc* + *R*3*c* coexistence phase to a complete rhombohedral (*R*3*c*) phase, indicated by the enlarged view of the characteristic peak fitting by using the *R*3*c* space group (Supplementary Fig. S2). Left shift and broad peak shape can be observed from the XRD patterns from BNT to LLM. Quantitative refined lattice parameters (listed in Table 1) show a slight increase in *a*, and decrease in *c*, indicating the less rhomboidity with a bigger volume that buildup the frame of LLM. Figure 1c shows the ferroelectric *P*-*E* hysteresis loops of BNT and LLM. The increased coercive field (*E*_C_) can be assigned to the fact that Mg atom enters B-site, like the behavior of “hard” PZT, even though the effect is considerably less pronounced. As observed from the SEM, it appears that there is an increase in grain size and the rectangular grain starts to take more spherical in LLM than in BNT. When Mg^2+^ incorporates in the octahedrally-coordinated perovskite B-site, the defect corresponding to oxygen vacancies is created for ionic charge compensation. Oxygen vacancies greatly promote the grain growth as they are favorable to the mass transport during sintering. Meanwhile, it has stronger pinning effect for the ferroelectric domain switching, thereby inducing a continuous increase in *E*_c_ and a slight decline in *P*_r_.

**Figure 2:**
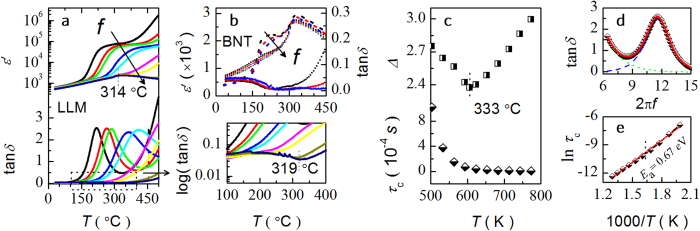
Temperature and frequency dependences of the dielectric properties. Dielectric permittivity *ε*’ and loss tangent tan*δ* of (**a**) LLM, at 100 Hz to 1 MHz with logarithmic increase in frequency, and (**b**) BNT at 1 kHz, 10 kHz and 100 kHz. The dot line in b refers to poled BNT. (**c**) Temperature dependence of relaxation intensity and relaxation time derived from the modified Debye equation based on tan*δ*-*f* plots. (**d**) Frequency dependence of the tan*δ* fitting for LLM. (**e**) The Arrhenius law describes the linear relationship between ln (*τ*_*c*_) and inverse temperature

Figure 2 provides dielectric permittivity *ε*’ and loss tangent tan*δ* of LLM and BNT as a function of temperature from RT to 500 °C. There are two characteristic dielectric anomalies in BNT (Fig. 2b). The frequency dispersion at ~200 °C indicates a thermal evolution of ferroelectric polar nanoregions (PNRs) of *R*3*c* and *P*4*bm*, followed by a relaxation of *P*4*bm* PNRs emerged from *R*3*c* PNRs, giving rise to the absolute maximum *T*_m _~ 320 °C. For the poled BNT, a clear *T*_d_ at ~178 °C is presented by the frequency-independent anomaly in the dielectric permittivity *ε*’ and tan*δ*, where the transition between the field-induced ferroelectric to relaxor takes place. However, the dielectric characteristics of the LLM are different from those of pure BNT and BNT-based solid solutions. Unlike the normal ferroelectrics and even most relaxors, the LLM exhibits a relatively high *ε*’ of ~10^5^ and strong frequency dispersion over a broad temperature range with a diffuse transition temperature. At high frequencies of >100 kHz, it demonstrates an anomaly at about 314 °C, in accordance with the changes in tan*δ* at 319 °C from the enlarged view of the tan*δ*-*T*. To investigate the relaxation mechanism in the intermediate temperature range (150–450 °C), the relaxation intensity (*Δ*) and relaxation time (*τ*_c_) are determined by fitting the tan*δ*-*f* curves with modified Debye model originated from equation (1).
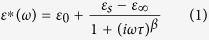


Here, *ε*_*s*_ is the static permittivity, *ε*_*∞*_ is the permittivity at high frequency, and *β* reflects the distribution width of the relaxation time. Fitting details are described elsewhere^13^. Figure 2d shows the well fitted temperature dependent tan*δ* using a nonlinear fitting method. Interestingly, an inflection point is observed at 333 °C from the temperature dependence of *Δ*. It seems that no obvious change is observed from the temperature dependent relaxation time, indicating the same relaxation mechanism. The linear relationship between the ln(*τ*_*c*_) and inverse temperature suggests the presence of a thermally activated process and the activation energy *E*_a_ is derived to be 0.67 *e*V described by the following equation,
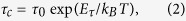
where *τ*_0_ is the pre-exponential relaxation time, *k*_*B*_ the Boltzmann constant, and *E*_*τ*_ the activation energy. Such an activation energy is much smaller than the typical BNT grain activation energy (~1.7 *e*V), which can be attributed to the motion of oxygen vacancies.

**Figure 3:**
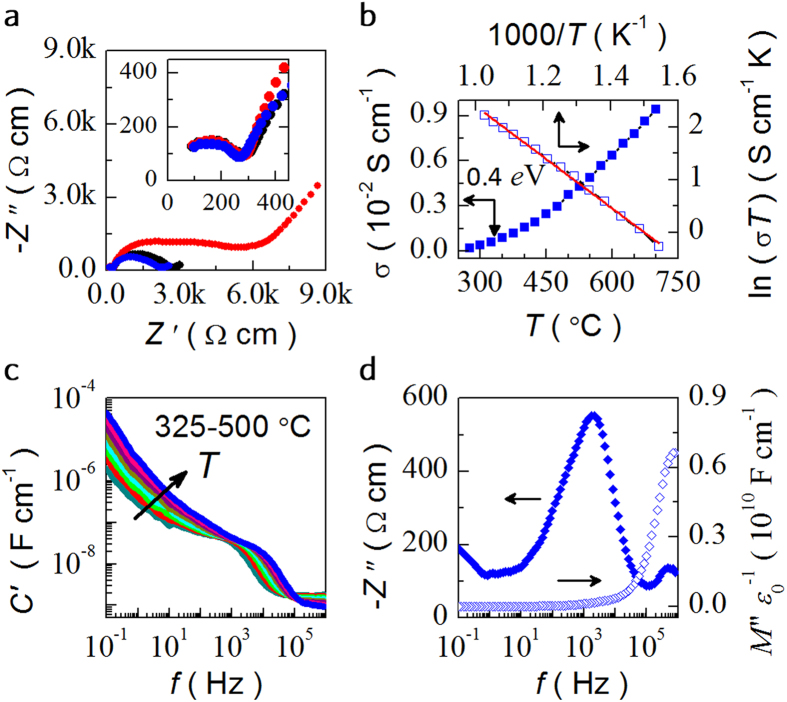
Ac impedance spectra and conductive properties of LLM. (**a**) Nyquist representations at 500 °C in oxygen (black), air (blue) and nitrogen (red), respectively. (**b**) Temperature dependence of electrical conductivity of LLM, which can be fitted with a conventional Arrhenius law in the linear part. Variation of (**c**) *C’*, (**d**) *Z*” and *M”* with frequency at selected temperatures for LLM.

To further explore the relaxation process, complex impedance spectra (Z*) are measured in different atmospheres. Typical impedance spectra of the LLM recorded at 500 °C are shown in Fig. 3. LLM exhibits two semicircular arcs in the frequency range (1 MHz–0.1 Hz), corresponding to the different contributors. The high-frequency semicircle can be attributed to bulk response, which is fitted with a parallel *RC* circuit and the capacitance is calculated to be 1.18 nF. The distorted intermediate arc can be ascribed to the grain boundary resistance. The low frequency spike represents the blocking electrode components, indicating the nature of ionic conduction. As observed in Fig. 3a, the high frequency arc associated with the bulk contribution is independent on the atmospheres of oxygen or nitrogen, which also supports the assumption that the material has ionic conduction characteristic in the measured oxygen partial pressure *P*_o2_. However, the arcs from the grain boundary and the electrode components of LLM display much *P*_o2_ dependence and show more resistive in nitrogen, suggesting the oxide ionic dominated process accounted as equation (3).
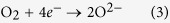


The low frequency intersection point of the semicircle with the *Z*’ axis is regarded as the bulk resistivity. Figure 3b shows temperature dependence of the bulk conductivity and the Arrhenius plot fitted with the equation *σT* = *σ*_0_exp (−*E*_a_/*k*_*B*_*T*), yielding an activation energy *E*_a_ = 0.4 *e*V. Compared with the previous report on Mg-doped and nonstoichiometric BNT^26^, the conductivity with temperature here increases monotonously and no noticeable inflection point is detected. Meanwhile, the grain boundary arc remains till 700 °C. The bulk conductivity in LLM is as high as ~0.01 S/cm, also indicating potential applications in solid oxide fuel cells (SOFCs). The ionic conduction characteristic is further illustrated by the ion blocking phenomena at electrode-sample interfaces with very high capacitance values at low frequencies (Fig. 3c). Two plateaus are attributed to the bulk and grain boundary response, respectively. Combined frequency dependent *Z*” and *M*” spectroscopic plots show that the *M*” plot contains a single peak, corresponding to the intermediate semicircle in Fig. 3a, whereas the *Z*” plot shows two peaks. The higher frequency *M*” peak represents a bulk component of the sample. The characteristic of overlapping *Z*” and *M*” peaks at high frequency signatures a long range process, which is associated with the ions diffusion between the O sites other than the localized relaxation.

**Figure 4:**
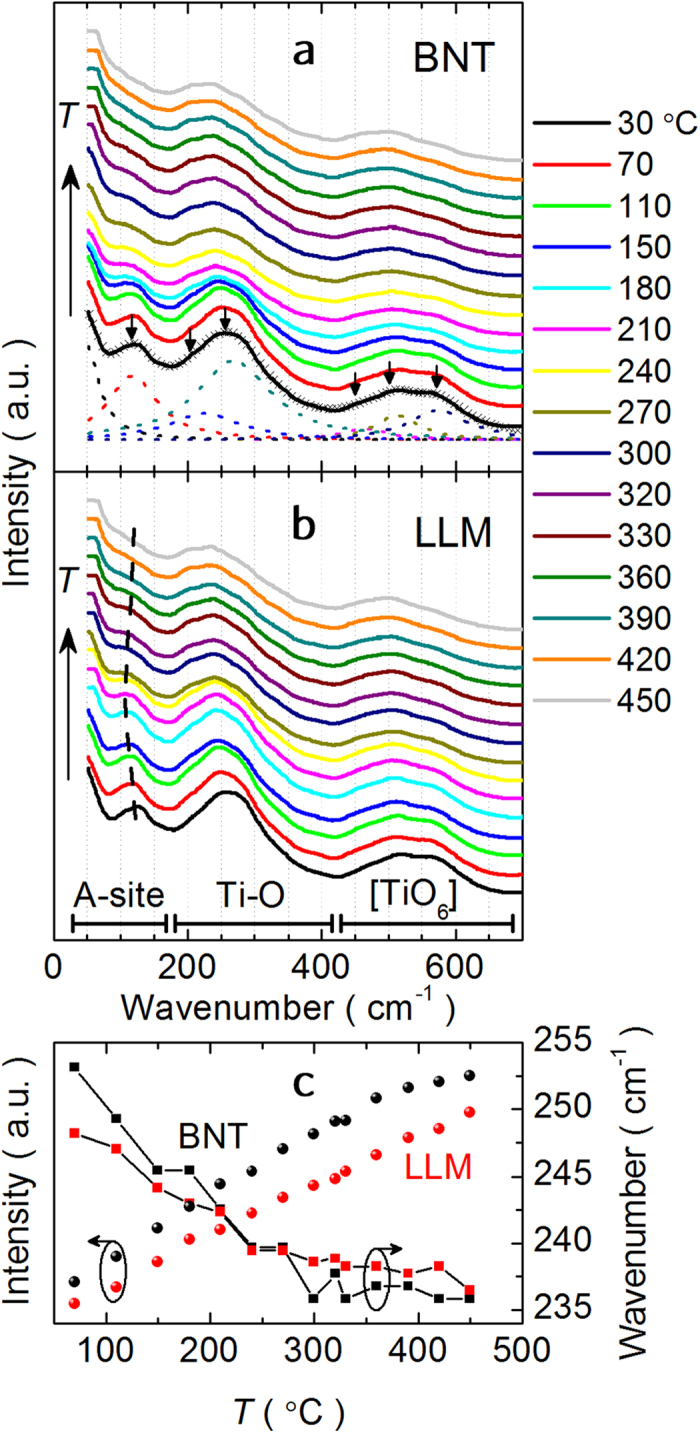
Temperature dependences of Raman properties. Temperature dependence of Raman spectra for (**a**) BNT and (**b**) LLM. (**c**) Raman intensity evolution with temperature at ~130 cm^−1^ mode normalized to that of the left peak valley at RT and wavenumber shift related to Ti-O vibration of BNT and LLM.

In order to identify the origin of the giant dielectric permittivity and the structural distortions in the phase evolution with temperature in BNT and LLM, temperature dependent Raman spectra from 30 °C to 450 °C is conducted with the spectral deconvolution into six Lorentzian-shape peaks from 100 to 700 cm^−1^. The modes vibrations that are responsible for the Raman plots are already available in the literature^28^,^29^. The Raman spectra and their evolution with the temperature in LLM are similar to those in BNT, indicated by (i) the weakening and softening mode at ~130 cm^−1^, then disappearing at elevated temperature, (ii) the broadening mode at ~250 cm^−1^, leaning by two remarkable shoulders gradually, and (iii) the broadening peak constituted by the three overlapping peaks with comparable intensity at 450–600 cm^−1^. Raman bands in both BNT and LLM are relatively broad as the temperature is increased due to the disorder on the random occupancy of cation A sites in the perovskite structure and the overlapping Raman modes. The spectral changes are consistent with a thermal induced structural transition from rhombohedral symmetry on average to tetragonality as the active Raman modes increase from 13 to 15 during the phase transition^30^,^31^. However, it is hard to unambiguously deduce where the key transition point takes place according to the temperature dependent Raman modes. Compared with the thermal evolution of Raman spectra in BNT, qualitative feature can be observed from the figure that the peak attributed to the Na-O vibrations at ~130 cm^−1^ changes more gently in LLM. This is further described by the distinct feature of the intensity change at ~130 cm^−1^ mode normalized to that of the left peak valley (Fig. 4c). Meanwhile, more moderate negative shift, which describes the reduction of the anion-cation displacements, is obtained for the wavenumber centered at 250 cm^−1^ as a function of temperature. Nevertheless, no distinct or discontinuous changes observed in the temperature induced Raman shift illustrates the symmetry change in the tilt system and the cation displacements.

**Figure 5:**
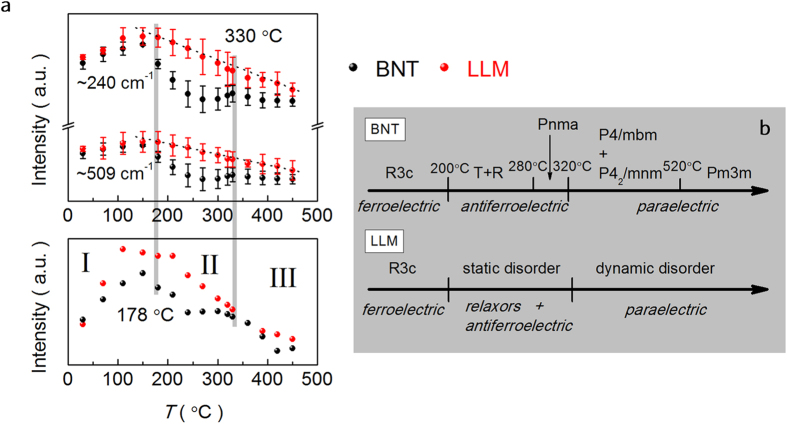
(**a**) Raman intensity and their relative intensity of *I*_240_/*I*_509_ evolution with temperature of BNT and LLM. (**b**) Proposed phase changes of the poled BNT and LLM with temperature.

The intensity variation of the modes, regarded as the main source of the relaxor features, reflects the ferroelectric state in perovskites through the coupling of light to the polarization in polar microregions^32^,^33^,^34^. It is expected to exhibit anomalies near the phase transition temperatures. The temperature dependent intensity of the characteristic modes at ~240 cm^−1^, ~509 cm^−1^ and the relative intensity of *I*_240_/*I*_509_ are displayed in Fig. 5. Interestingly, the data evolution with temperature in BNT indicates two discontinues shifts and can be divided into three regions, which shows close relationship to the result of BNT, as demonstrated by Trolliard *et al.* in learning the thermal induced phase transition by electron diffraction^35^,^36^. The state of the poled BNT is proposed with the phase changes summarized in Fig. 5b. Thus, the valley in the intermediate temperature range of Raman intensity curve results from a competition between different phases, i.e., rhombohedral (*R*3*c*) (*R*) and tetragonal (*P*4*bm*) (*T*), *T* and orthorhombic (*Pnma*) (*O*). The deviation of the phase transition temperatures between the dielectric and Raman intensity anomalies is due to the diffuse characteristic of the dielectric response. In single crystal and ceramics of BNT, they also exhibit higher ferroelectric to relaxor transition temperature (*T*_F–R_) in dielectric plots^32^. Moreover, the overlapping modes cannot be precisely identified for their diffuse nature in Raman spectra.

**Figure 6:**
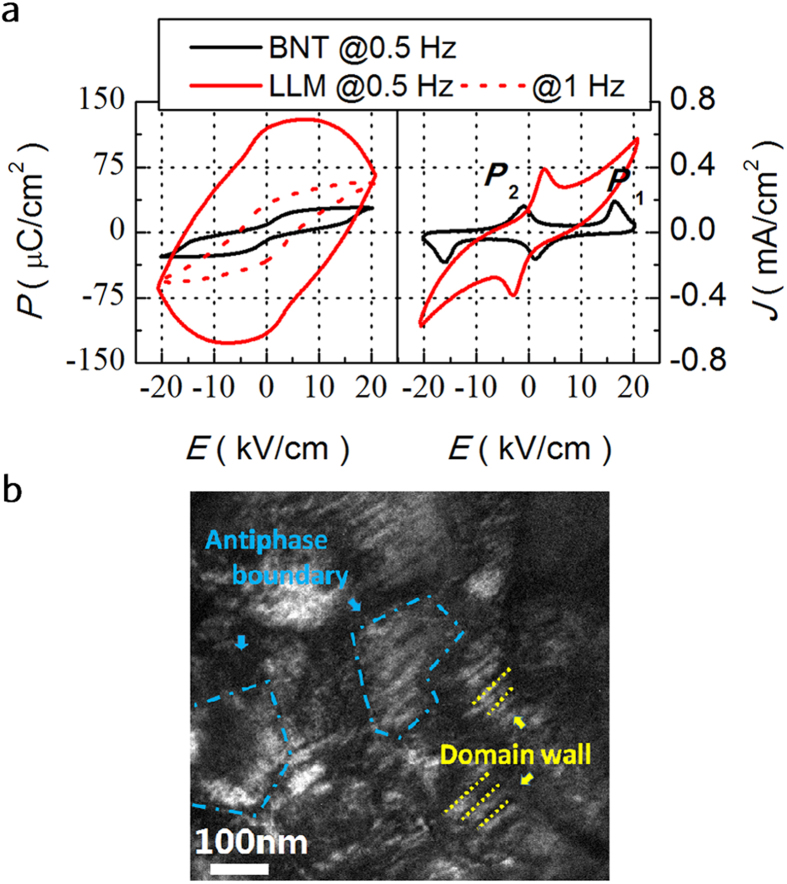
(**a**) *P*-*E* and *I*-*E* loops for BNT and LLM at selected frequencies at 181 °C. (**b**) Dark field images of the LLM sample with ferroelastic domain walls and antiphase boundaries.

Inspection of LLM reveals the similar tendency in Region I in which the intensity is decreased considerably upon cooling. That means that similar features associated with the relaxor and ferroelectric phase transition happen between Region II and I, where the nano-polar region would be frozen at the lower temperature. In that case, the LLM should stay in a ferroelectric state at ambient temperature in spite of the high ionic mobile charge embedded in the crystal matrix at high temperature. Figure 6a shows *P*-*E* and *I*-*E* loops at selected frequencies for BNT and LLM at 181 °C. BNT exhibits a pinched loop and four peaks on the corresponding *J*-*E* curve at 0.5 Hz, which is attributed to the reversible electric-field-induced transition between relaxor and ferroelectric^37^. Unlike BNT, the LLM also exhibits a pinched but a little “swelled” *P*-*E* loop at 0.5 Hz. The corresponding *J*-*E* curve shows two peaks. Again, the two peaks are attributed to backward switching from relaxor to ferroelectric phase as the two forward switching peaks are covered by the high leakage current. For BNT, the ferroelectric transforms back to relaxor phase as the applied electric-field is decreased. For LLM, as 181 °C is lower than *T*_d_ (~190 °C), a negative electric-field has to be applied to induce the phase transition. Moreover, the *P*-*E* loop of LLM at 1 Hz is “shrunken”. The leakage component is decreased since the mobility of the oxygen vacancy falls behind the high frequency.

In the high temperature region, their intensity of the Raman modes of LLM gradually lost and present a nearly linearity without discontinuity. No evident phase transition is observed although the reversible electric-field-induced transition between relaxor and ferroelectric does exist. In the whole level, an order-disorder transition is expected rather than the phase transition. Thus, we propose that LLM global structure at intermediate temperature exhibits a static disorder in the averaged cell (Fig. 5b), in analogy to the typical oxide ionic conductor La_2_Mo_2_O_9_, where the cubic-monoclinic phase transition is suppressed by the substitutions with a static oxygen disorder below the original phase transition temperature^38^,^39^. The introduction of Li^+^, La^3+^ and Mg^2+^ with stable chemical valence in BNT builds up the basic frame in which it facilitates the delocalized oxide conduction and somewhat suppresses the reduction of Ti^4+^ to Ti^3+^. The significant relaxation in temperature dependent large dielectric permittivity is mainly ascribed to the oxygen diffuse anomalies between different sites with special relaxation time *τ*. The oxygen vacancies will not be easy to be trapped and the cation displacement remains decoupled from tilting. The dielectric plots with temperature show a similar feature to that of La_2_Mo_2_O_9_^13^. In the same way, the Ga^3+^ replacing Mg^2+^ in BNT can also lead to abnormally high dielectric permittivity. As discussed above, LLM possesses delocalized conduction of oxygen ions, which makes the atom rearrangement easy. Highly disorder defect structure with a complex arrangement starts to fix and form limited size of ferroelectric order below the temperature at which a freezing process starts. Meanwhile, the domain nucleation density is greatly raised for the much higher concentration of oxygen vacancy. As a result, a higher density of domain walls and anti-phase boundaries are observed at ambient temperature in the ferroelectric state of LLM (Fig. 6b).

**Figure 7:**
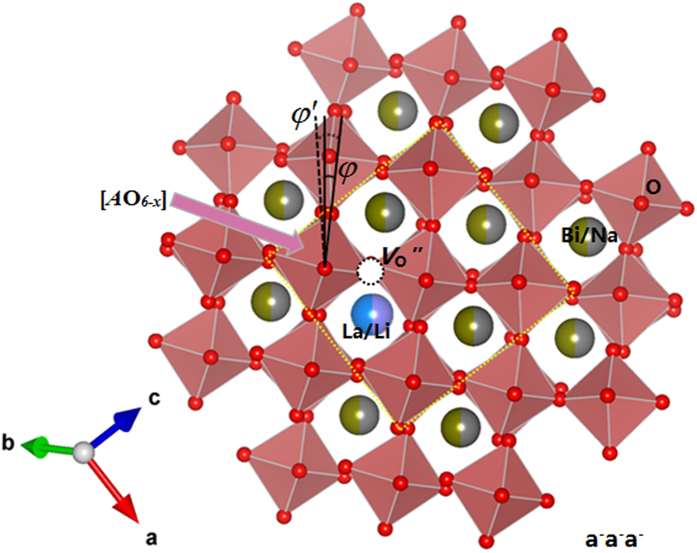
Schematic of the a^−^a^−^a^−^ tilt system seen in poled LLM rhombohedral perovskites arising from oxygen octahedral tilting about their threefold pseudo-cubic axes. The projection of the rhombohedral structure down [001]p. The yellow square frame represents the [100] and [010] direction of cubic cell, respectively. In the hexagonal setting of the *R*3*c* structure, the spontaneous polarization direction of [111] cubic peak is parallel to the c axis.

As discussed above, the poled LLM exhibits a pure rhombohedral structure and displays well peak splittings in its ferroelectric state. The peak splitting seems less obvious when compared LLM with BNT associated with all the peak left shift. Figure 7 shows the schematic of the a^-^a^-^a^-^ tilt system seen in poled LLM rhombohedral perovskites according to the refined data. In this phase the Na/Bi sites and Ti sites are displaced parallel to each other along [111] to give a polar ferroelectric phase. The oxygen octahedral gives rise to partial oxygen vacancy, marked as [*A*O_6−*x*_], from the low valance acceptor doping on Ti sites. The La^3+^ and Li^+^ atoms are introduced as the obstructer from the oxygen sites depletion by Bi^3+^ with lone-pair electron. Thus it leads weak bonded between the generated oxygen vacancies and Li/La sites. This can be described by equation (4) and (5), or equation (4) and (6).
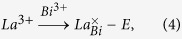

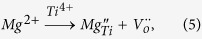

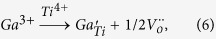
where *E* represents the oxygen sites depletion by lone-pair electron. As a result, the LLM structure owns a bigger free volume that facilitates the anomalous oxygen sites relaxation. Meanwhile, the octahedral tilt is reduced with the angle *φ* changing from 8.14° (BNT) to 6.34° (LLM). The polarization direction on the nanoscale in LLM deviates from the global polarization direction with enhanced static substitutional disorder at its ferroelectric state. The Ti sites related to the nearest oxygen sites on the average become non-equidistant and go backward against the possible oxygen vacancies after poling (Supplementary Fig. S3).

**Figure 8:**
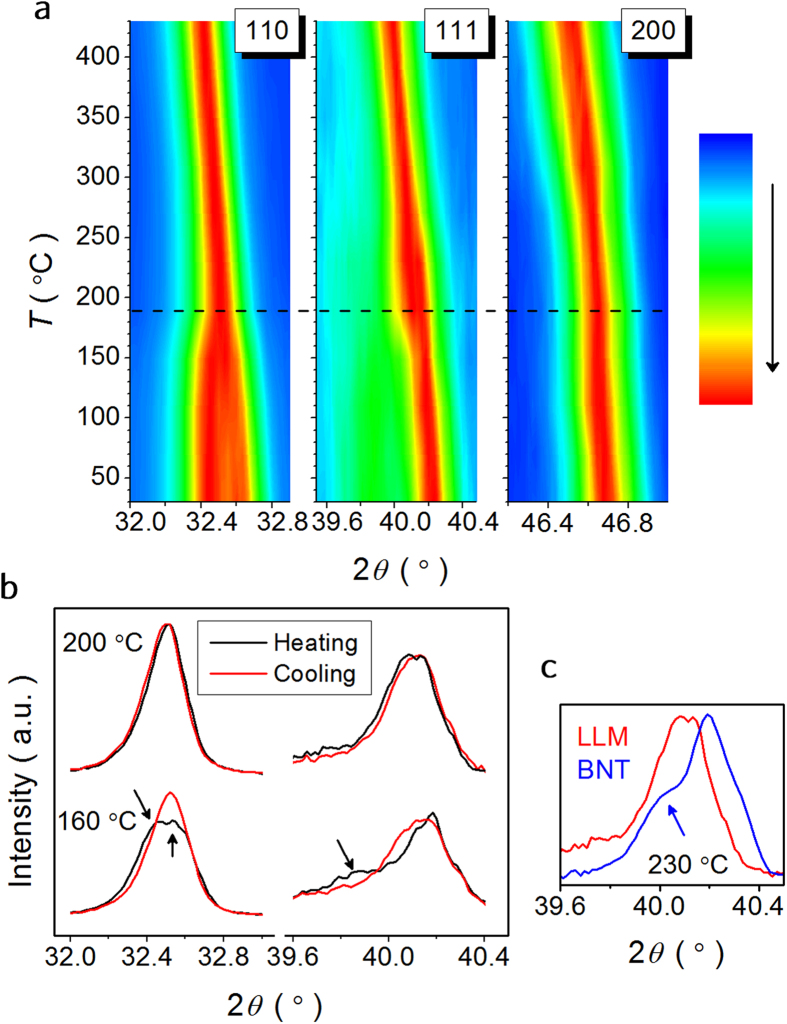
Temperature-controlled X-ray diffractograms of the poled LLM powder. (**a**) XRD patterns upon heating from 30 °C to 430 °C. (**b**) XRD patterns at 200 °C and 160 °C, (above and below *T*_F-R_), of the poled LLM at the heating and subsequently cooling process. (**c**) {111}_*c*_ peak of the poled LLM and BNT at 230 °C upon heating.

In order to further learn the phase transition with the temperature in special LLM, temperature dependent X-ray diffractograms performed on poled-crushed LLM powder are conducted. Figure 8a shows the evolution of selected Bragg peaks upon heating from 30 °C to 430 °C. In the contour plots, we observe a sudden change in both {110}_*c*_ and {111}_*c*_ peak of the poled LLM at ~190 °C, in agreement with the *T*_F-R_ in the dielectric and Raman anomalies. The field-stabilized homogeneous *R*3*c* structure seems to sustain till 190 °C, at which the splitting of {110}_*c*_ appears to collapse and the asymmetric {111}_*c*_ peak becomes symmetric above this temperature. Figure 8b displays XRD patterns of the poled LLM upon heating and subsequently cooling above and below *T*_F-R_. The inspection of the peak shape reveals the monoclinic distortion below the *T*_F-R_, since the LLM is regarded to be depoled after the heating treatment. In the intermediate temperature range, no drastic change besides the nearly single broad {111}_*c*_ peak is detected above the *T*_F-R_, indicating that the partial structural transition is suppressed for the high temperature anomalies. For comparison, XRD patterns of the poled LLM and BNT at 230 °C are provided in Fig. 8c. The {111}_*c*_ peak asymmetry survives above the *T*_F-R_ in BNT, as indicated by the arrow, since this pattern feature in intermediate temperature range in BNT is composed of a modulated structure according to the *R*3*c* + *P*4*bm* model^21^,^35^,^36^.

In summary, the dielectric properties of A/B sites co-substituted Bi_0.5_Na_0.5_TiO_3_ (Bi_0.5−*x*_La_*x*_Na_0.5−*x*_Li_*x*_Ti_1−*y*_*M*_*y*_O_3_ (*M* = Mg^2+^, Ga^3+^)) are characterized in a broad range of temperature and frequency. The giant dielectric permittivity significantly varies from 3*10^3^ to 10^5^ under the inhomogeneity with the conductive grain and resistive grain boundary responses. The diffuse anomalies with step-like frequency dispersion is a thermal activated intrinsic process, which originated from the substitutional disordered Bi/Na sites. The phase transition at ~330 °C is suppressed and the relaxor to ferroelectric phase transition is still clearly observed at ~190 °C. Structural changes in the cation displacement and the oxygen octahedral tilt are indicated in this perovskite by analyzing the composition and temperature dependence of XRD patterns and Raman spectra.

## Methods

### Materials synthesis

Polycrystalline Bi_0.5−*x*_La_*x*_Na_0.5−*x*_Li_*x*_Ti_1−*y*_*M*_*y*_O_3_ (*M* = Mg^2+^, Ga^3+^) employed were prepared by using a conventional solid state reaction method, with mixing appropriate amount of high purity La_2_O_3_ (99.99%), Bi_2_O_3_ (99.9%), Na_2_CO_3_ (99.8%), Li_2_CO_3_ (99.99%), TiO_2_ (98%), MgO (99.99%) and Ga_2_O_3_ (99.999%) as raw powders. The powders were weighed and mixed by ball milling in isopropyl alcohol for 12 h. The mixtures were calcined at 800 °C for 4 h. The calcined powders were remilled for 12 h, and then cold isostatically pressed into pellets of 10 mm in diameter at a pressure of 250 MPa. The pressed pellets were sintered at 1125–1175 °C embedded in precursor powders to avoid Bi volatilization. Sliver electrodes were coated on both the polished surfaces of the resulting pellets and fired at 850 °C for 30 min for electric test.

### Structural characterization

Phase structures of the powders from the unpoled and poled pellets were investigated by X-ray diffraction (XRD; XRD-7000, Shimadzu, Kyoto, Japan) with Cu K_α_ radiation. Long run diffractograms for structure determination were collected in the Bragg angle (2*θ*) range from 10° to 130°. The Rietveld program Fullprof was used for full-pattern matching and structural refinements at ambient temperature. High temperature XRD (2*θ*, 10°–80°) was conducted to investigate the phase transition ranging from RT to 430 °C, then cooling to RT. A stabilization time of 10 min was systematically applied between each measurement. Morphological images were obtained by scanning electron microscope (SEM; JSM-5610, JEOL, Tokyo, Japan). The TEM images were obtained from the as-sintered pellets by using a transmission electron microscopy (TEM; Tecnai F30, FEI, Hillsboro, OR, USA). Raman spectra were recorded on polished sintered pellets using 514.5 nm excitation by a Jobin-Yvon LabRam HR800 (Horiba Jobin Yvon Inc., Paris, France) from 30 °C to 450 °C with 20 min of thermal stabilization on each measurement.

### Electric properties measurements

Dielectric properties were measured by using a precision impedance analyzer (4294A, Agilent, Santa Clara, CA, USA) from RT to 500 °C. Impedance spectra were collected by a Schlumberger Solartron SI 1260 frequency response analyzer from 800 °C to 300 °C in oxygen, air and nitrogen, respectively. Electric-field-induced polarization curves were collected by using a ferroelectric test unit (TF-2000, aix-ACCT, Aachen, Germany) from 30 °C–190 °C.

## Additional Information

**How to cite this article**: Liu, X. *et al.* Origin of anomalous giant dielectric performance in novel perovskite: Bi_*0.5-x*_La_*x*_Na_*0.5-x*_Li_*x*_Ti_1-*y*_M_*y*_O_3_ (*M*=Mg^2+^, Ga^3+^). *Sci. Rep.* **5**, 12699; doi: 10.1038/srep12699 (2015).

## Supplementary Material

Supplementary Information

## Figures and Tables

**Table 1:** Refined parameters resulting from the *R*3*c* refinement of the poled BNT and LLM at RT.

	**Symmetry**	***a**, **b*** **(Å)**	***c*** **(Å)**	**Volume**	**Profile fit**
BNT	*R*3*c*	5.4780	13.5503	352.1394	*R*_p_ 4.8 χ^2^ 2.74
LLM		5.4837	13.5479	352.8159	*R*_p_ 5.23 χ^2^ 4.04
