# Supplementary material for: Origin of anomalous giant dielectric performance in novel perovskite: Bi0.5−xLaxNa0.5−xLixTi1−yMyO3 (M = Mg2+, Ga3+)
Source: Sci Rep. 2015 Aug 4;5:12699. doi: 10.1038/srep12699 (PMC4523863; doi:10.1038/srep12699)
Supplement: Supplementary Information [file srep12699-s1.pdf]

Supporting Information for

Origin of anomalous giant dielectric performance in novel perovskite: $Bi_{0.5-x}La_xNa_{0.5-x}Li_xTi_{1-y}M_yO_3$ (M=Mg^{2+}, Ga^{3+})

Xiao Liu, Huiqing Fan*, Jing Shi & Qiang Li

State Key Laboratory of Solidification Processing, School of Materials Science and Engineering, Northwestern Polytechnical University, Xi'an 710072, China

*Correspondence and requests for materials should be addressed to H.F. (hqfan3@163.com)

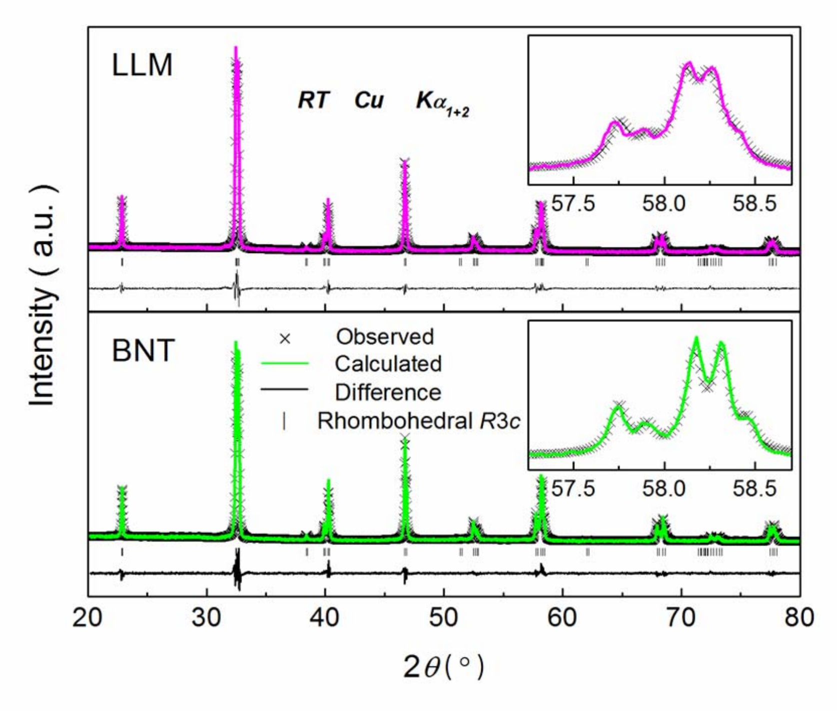

Figure S1 | Selected 2θ range of Rietveld fitting for the long-time running powder X-ray diffraction patterns of poled BNT and LLM, which are refined as rhombohedral cell (s. g. *R*3c). The insets show the enlarged view of $(211)_{pc}$ pseudocubic peak. The fitting can fully describe the measured diffraction patterns with better quality. The peak splitting seems less obvious when compared LLM with BNT associated with all the peak left shift. This indicates that LLM possesses bigger cell volume with lower symmetry of *R*3*c* ordering.

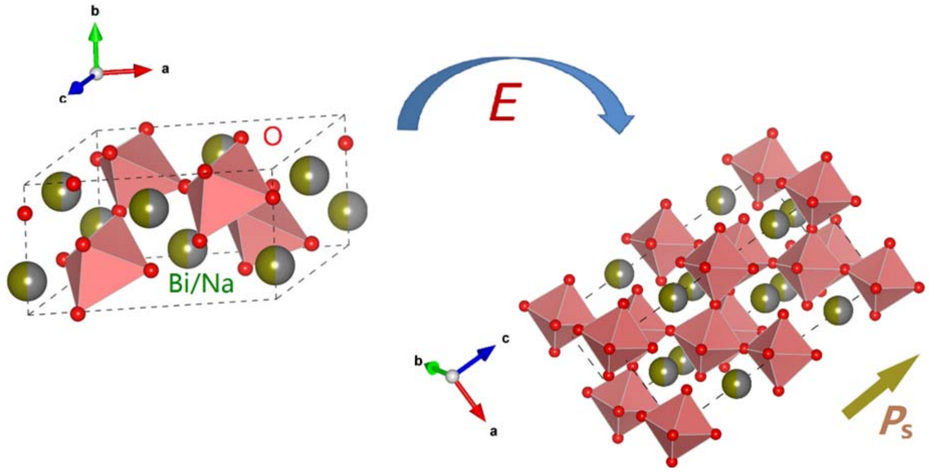

Figure S2 | LLM and poled LLM structure refined by monoclinic *Cc* and rhombohedral *R*3*c* model respectively. The monoclinic unit cell consists of four formula units and the rhombohedral of six. When given a poling electric field *E*, the phase can be described more accurately by the *R*3*c* structure as it exists irreversible relaxor to ferroelectric phase transition induced by *E*. In the hexagonal setting of the *R*3*c* structure, the spontaneous polarization direction of [111] cubic peak is parallel to the c axis as shown.

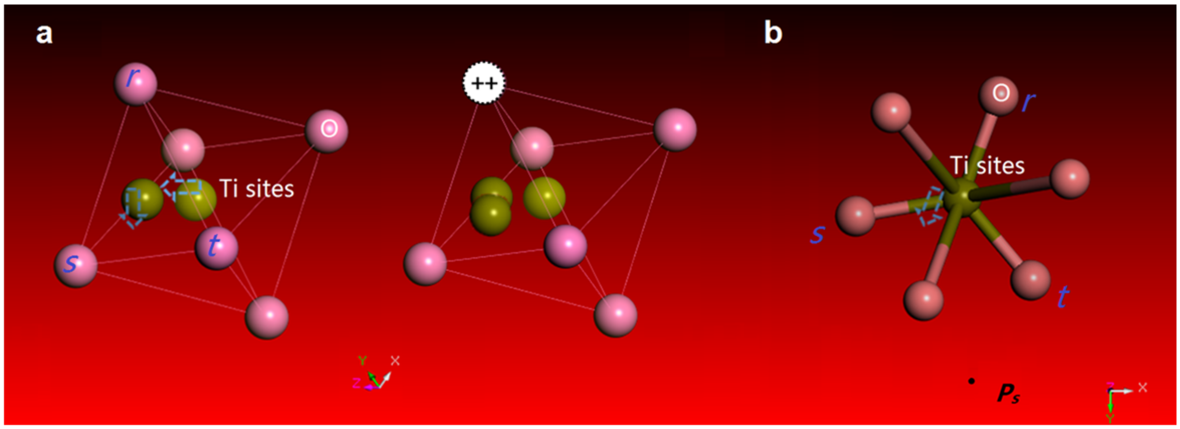

Figure S3 | Schematic of oxygen octahedral [TiO_{6-x}] titling and Ti-sties displacement of ABO_3 unit with different view. The original polarization direction associated with the Ti sites displacements of BNT is along the z axis in the left of **a**. The extra oxygen vacancies caused by acceptor doping on Ti sites in LLM leads to the electro-positivity of the corresponding oxygen sites (**b**). As the weak bond between the non-lone pair containing La atom and oxygen vacancy, the bond distance of the vacancy and Ti sites becomes large with the sites displacing along the opposite direction in *rst* plane as shown. In **b**, the average polarization of [111] is perpendicular to the surface.

The *r*, *s*, *t* positon related to Ti sites are almost equidistant in BNT, while only the *s*, *t* to Ti sites are equidistant in LLM according to the refined results. As a result, the LLM structure owns a bigger free volume that facilitates the anomalous oxygen sites relaxation. The polarization direction on the nanoscale in LLM is likely to deviate from the average polarization direction with enhanced static substitutional disorder at its ferroelectric state.

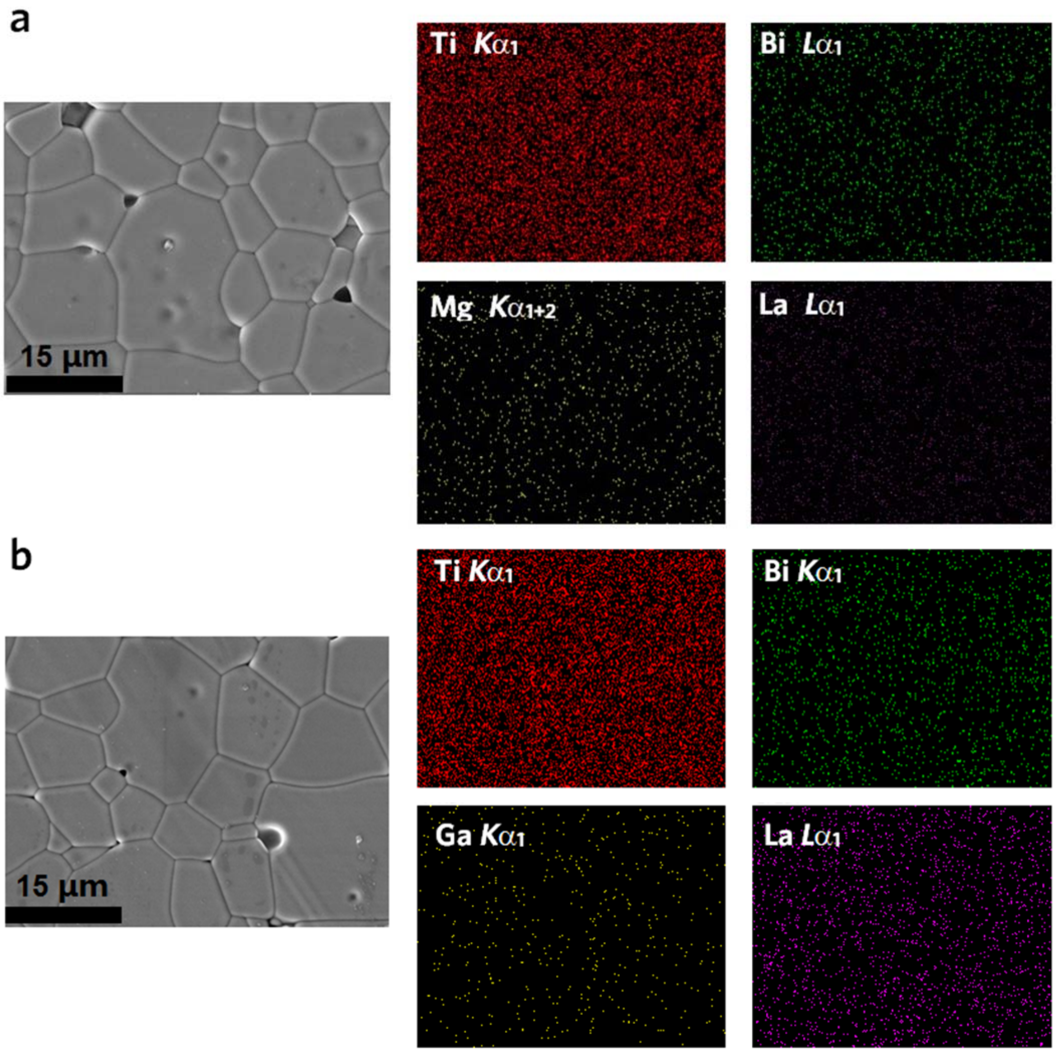

Figure S4 | SEM micrographs of selected samples. All were polished and thermally etched at 1050 ^{o}C for 30 min prior to SEM. **a**, LLM **b**, $Bi_{0.49}La_{0.01}Na_{0.49}Li_{0.01}Ti_{0.99}Ga_{0.01}O_3$ (LLG). All samples exhibit high density and big grain size with the uniform distribution of corresponding element. By contrast, it contains a little richer in Mg and Ga elements near the grain boundary. Oxygen vacancies induced by the acceptor doping greatly promote the grain growth, as they are favorable to the mass transport during sintering compared with other BNT based ceramics.
